# Investigation of Pathogenic Genes in Peri-Implantitis from Implant Clustering Failure Patients: A Whole-Exome Sequencing Pilot Study

**DOI:** 10.1371/journal.pone.0099360

**Published:** 2014-06-12

**Authors:** Soohyung Lee, Ji-Young Kim, Jihye Hwang, Sanguk Kim, Jae-Hoon Lee, Dong-Hoo Han

**Affiliations:** 1 Department of Prosthodontics, Oral Science Research Center, College of Dentistry, Yonsei University, Seoul, Korea; 2 Department of IT Convergence and Engineering, Pohang University of Science and Technology, Pohang, Korea; 3 Department of Prosthodontics, Oral Science Research Center, College of Dentistry, Yonsei University, Seoul, Korea; Nazarbayev University, Kazakhstan

## Abstract

Peri-implantitis is a frequently occurring gum disease linked to multi-factorial traits with various environmental and genetic causalities and no known concrete pathogenesis. The varying severity of peri-implantitis among patients with relatively similar environments suggests a genetic aspect which needs to be investigated to understand and regulate the pathogenesis of the disease. Six unrelated individuals with multiple clusterization implant failure due to severe peri-implantitis were chosen for this study. These six individuals had relatively healthy lifestyles, with minimal environmental causalities affecting peri-implantitis. Research was undertaken to investigate pathogenic genes in peri-implantitis albeit with a small number of subjects and incomplete elimination of environmental causalities. Whole-exome sequencing was performed on collected saliva samples via self DNA collection kit. Common variants with minor allele frequencies (MAF) > = 0.05 from all control datasets were eliminated and variants having high and moderate impact and loss of function were used for comparison. Gene set enrichment analysis was performed to reveal functional groups associated with the genetic variants. 2,022 genes were left after filtering against dbSNP, the 1000 Genomes East Asian population, and healthy Korean randomized subsample data (GSK project). 175 (p-value <0.05) out of 927 gene sets were obtained via GSEA (DAVID). The top 10 was chosen (p-value <0.05) from cluster enrichment showing significance of cytoskeleton, cell adhesion, and metal ion binding. Network analysis was applied to find relationships between functional clusters. Among the functional groups, ion metal binding was located in the center of all clusters, indicating dysfunction of regulation in metal ion concentration might affect cell morphology or cell adhesion, resulting in implant failure. This result may demonstrate the feasibility of and provide pilot data for a larger research project aimed at discovering biomarkers for early diagnosis of peri-implantitis.

## Introduction

The successful incorporation of titanium implants in the rehabilitation of edentulous patients has been well-investigated over the years [1]. According to statistics, over 2 million dental implants are installed annually, a number that could rise over the next few years. Despite the high success rates of osseointegrated implants, increasingly frequent biological complications related to implants often result in implant loss [2]–[4]. The failure rate of dental implants as currently reported is approximately 3–5% within the first 10 years [5], [6]. Peri-implantitis is a biological complication that occurs in dental implant patients and comprises a range of destructive inflammatory processes affecting surrounding soft and hard tissues for which there is no current gold-standard treatment [7]. Diagnosis is based on changes of color in the gingival, bleeding and probing depth of peri-implant pockets, suppuration, X-ray, and gradual loss of bone height around the tooth [8]. Despite all these methods, we need to note that peri-implantitis can develop without any obvious symptoms such as pain [9], [10] so that patients often fail to notice the development of the disease. Due to difficulties in early detection of peri-implantitis, implant failures are steadily increasing, with clinical studies reporting near 4% implant loss [11]–[13]. Hence, we need a fresh approach for diagnosing peri-implantitis.

Previous studies have found that peri-implantitis and implant failures cluster in subsets of individuals and that a patient who has lost one implant is at elevated risk of experiencing other implant losses [14], [15]. Such failure of more than one implant in a patient, not necessarily in the same area or quadrant, is called implant clusterization failure. In a previous literature review, findings suggested that implant failures are not randomly distributed in the treated populations, but rather cluster in specific high risk groups and individuals [16]. Peri-implantits pathogenesis research has faced the challenge of multiple casualties affecting the disease. Both multiple genes and environmental factors may play a critical role in peri-implantitis. Patients with clusterization failure were carefully chosen for the current study based on having a distinctive phenotype of the disease, a relatively healthy lifestyle, and similar environmental traits. The cluster phenomenon supports evidence that specific host characteristics, including genetic factors, play an important role in bone resorption and in the development of peri-implantitis leading to implant failure [17]. Therefore, early diagnosis of peri-implantitis through the detection of pathogenic genes in advance of visual symptoms and radiographic findings may prevent implant failure due to severe peri-implantitis and increase the implant success rate as well.

There have been several studies on the role of cytokines in peri-implantitis [18]. A recent systematic review of the association between genetic predisposition and biological complications of dental implants suggested that there is no strong association among specific genetic polymorphisms (IL-1A, IL-1B, IL-17RC) and peri-implantitis, although there was a notable tendency indicating a link between the IL-1 genotype and peri-implantitis [19], [20]. Another study presented MMP-8 and PGE-2, both regulated by IL-1 as possible genetic markers for unsuccessful implants based on their role in regulating the extra cellular matrix (ECM) which may enhance bone healing within defects and promote implant osseointegration [21]. Nonetheless, no prior studies have established a genetic association with peri-implantitis, supporting the need for a new genetic association study.

Whole Exome Sequencing (WES) in genetics has had the greatest impact on Mendelian disorders, distinguishing more than 100 genes in rare Mendelian disorders between 2010 and 2012 [22]. Approaches in investigating novel genetic mechanisms, phenotypic variability, modifier genes, allelic variants, and genetic variations in Mendelian disorders may also help elucidate complex disorders such as peri-implantitis. Unlike Mendelian disorders, peri-implantitis is a multi-factorial disorder involving complex disorders, multiple genes, as well as lifestyle or environmental factors. To maximize the specificity of our results, we selected patients with clusterization failure due to peri-implantitis. WES alone may not provide pragmatic results on the association between a specific disease and genetic variants due to the extensive raw data, pointing out the need to incorporate bioinformatic data management, computational analyses, and data mining. Incorporation of Gene Set Enrichment Analysis (GSEA) and a protein functional network study of WES data may determine genetic variants explicitly related to peri-implantitis.

The objective of this study is to find pathogenic genes associated with peri-implantitis via WES, GSEA, and network analysis.

## Materials and Methods

### Ethics Statement

All research involving human subjects or human data was approved by the Institutional Review Board of Yonsei University College of Dentistry (Yonsei IRB No. n2-2012-0023). All clinical investigation was performed in accordance with the Declaration of Helsinki. Written informed consent was obtained from all participants before enrolling in this study.

### Patient Selection

Six individuals with clusterization failure due to severe peri-implantits aged between 50 and 68 were analyzed by massively parallel sequencing in this study (1 male, 5 female).

The six individuals had been treated with osseointegrated titanium implants for partial or complete edentulism in the Implant Clinic of Yonsei University Dental Hospital from November 2002 to October 2013. These patients had no history of parafunctional habits, smoking experience, periodontal disease, or systemic disease such as diabetes mellitus and osteoporosis. The number of implants inserted to the patients was 55, 26 of which were explanted. Presence of mobility, vertical resorption of less than 50%, and pus discharge on patients yielded a poor prognosis in all selected cases.

### Comparing Data Set

126 Koreans from the GSK project aged between 23 and 46 (109 male, 17 female) were chosen as randomized subsample from the reference population along with dbSNP137 and the 1000 Genomes East Asian population. Exome data of the Korean randomized subsample, dbSNP137, and 1000 Genomes East Asian population were used to eliminate common variants from the six selected patients' WES data. The 126 Korean randomized subsamples, healthy Koreans regardless of gender and age, had originally been recruited for a thyroid cancer study (GSK project). This group had no history of diseases known to affect periodontal disease, such as diabetes and osteoporosis.

### Sample Collection

Each patient's saliva was collected using self DNA collection kit. Self DNA collection kit instructions were followed: first, all 6 individuals were asked to collect 2 mL of saliva in the tube of an Oragene DNA Self-Collection kit containing 2 mL of DNA-preserving solution. The lid was closed to release the storing liquid to mix with the saliva. Genomic DNA collection, DNA extraction, and further analysis were performed by DNA Link Inc., Seoul, South Korea.

### Whole Exome Sequencing on HISEQ 2000 using SureSelect All Exon kit 50 Mb

With an OD260/280 ratio of 1.8–22, DNA should be as intact as possible. Quality of DNA was checked by 1% agarose gel electrophoresis and PicoGreen dsDNA Assay. SureSelect sequencing libraries were prepared following the manufacturer's instructions using a Bravo automated liquid handler. One ug of genomic DNA in 120 mL EB buffer was fragmented to a median size of 150 bp using a Covaris-S2 with the following settings: duty cycle 10%, intensity 5, cycles per burst 200, and mode frequency sweeping for 360 s at 4°C. Capillary electrophoresis on DNA 100 chips was used to evaluate the efficiency of the fragmentation. Following the manufacturer's protocol, sequencing adapters were ligated on the DNA fragments. PCR was used to amplify the adapter ligated DNA. Capillary electrophoresis was used to ensure the quality of the PCR products. In preparing the hybridization buffer, #1, #2, #3, and #4 reagents were mixed. The amplified DNA fragments were concentrated to 500 ng in 3.4 ul. The 500 ng of DNA was mixed with SureSelect block #1, #2, and #3 reagents. The hybridization buffer and DNA blocker mix were incubated for 5 min at 95°C followed by 10 min incubation at 65°C in a thermal cycler. Rnase block was added to the SureSelect oligo capture library and the capture library was incubated for 2 min at 65°C. In a thermal cycler, the hybridization buffer followed by the DNA blocker mix was added to the capture library and the mixture was incubated for 24 hours at 65°C. Fifty ul of streptavidin coating the Dynal MyOne Streptavidin T1 were washed three times with 200 ml SureSelect binding buffer and resuspended in 200 ml of the binding buffer. After being added to the bead suspension, the hybridization mixture was incubated for 30 min at room temperature with mixing. The beads were washed with 500 mL SureSelelct wash buffer #1 for 15 min at room temperature followed by three times wash with 500 mL SureSelect buffer #2 for 10 min at 65°C and NDA was eluted with 50 mL SureSelect elution buffer for 10 min at room temperature. Fifty mL of SureSelect neutralization buffer was added to the eluted DNA. Purification of the reaction product was done with the AMPure XP bead. Using Herculase II Fusing DNA Polymerase, the captured library was amplified to add index tags, capillary electrophoresis then being used to verify the quality of the amplified libraries.

The 6 libraries, index tagged in equimolar amounts in the pool, were combined after GPCR using SYBR Green PCR Master Mix. Cluster generation appeared in the flow cell on the cBot automated cluster generation system and the flow cell was loaded on the HISEQ 2000 sequencing system for sequencing with 2×101 bp read length.

### Whole Exome Sequencing and variant analysis

On average, 5.96 gigabases of raw sequence were generated per sample to achieve an average of 53.18× coverage of the WES target regions (51 megabases). The 6 individuals' raw sequencing data was screened for common artifacts prior to comparison with control data sets. SNP variants were selected and filtered according to the following criteria. Variants were considered to be common if present in the 1000 Genomes Project East Asian database. Variants present in 1000 Genomes Project, dbSNP137, and 126 Korean Population data were filtered out from case variants. Variants were eliminated if they had minor allele frequencies (MAF) > = 0.05 from all control datasets used for comparison. Effect, impact (high or moderate), and loss of function in WES were established with SnpEff v3.3 h (http://snpeff.sourceforge.net/).

**Figure 1:**
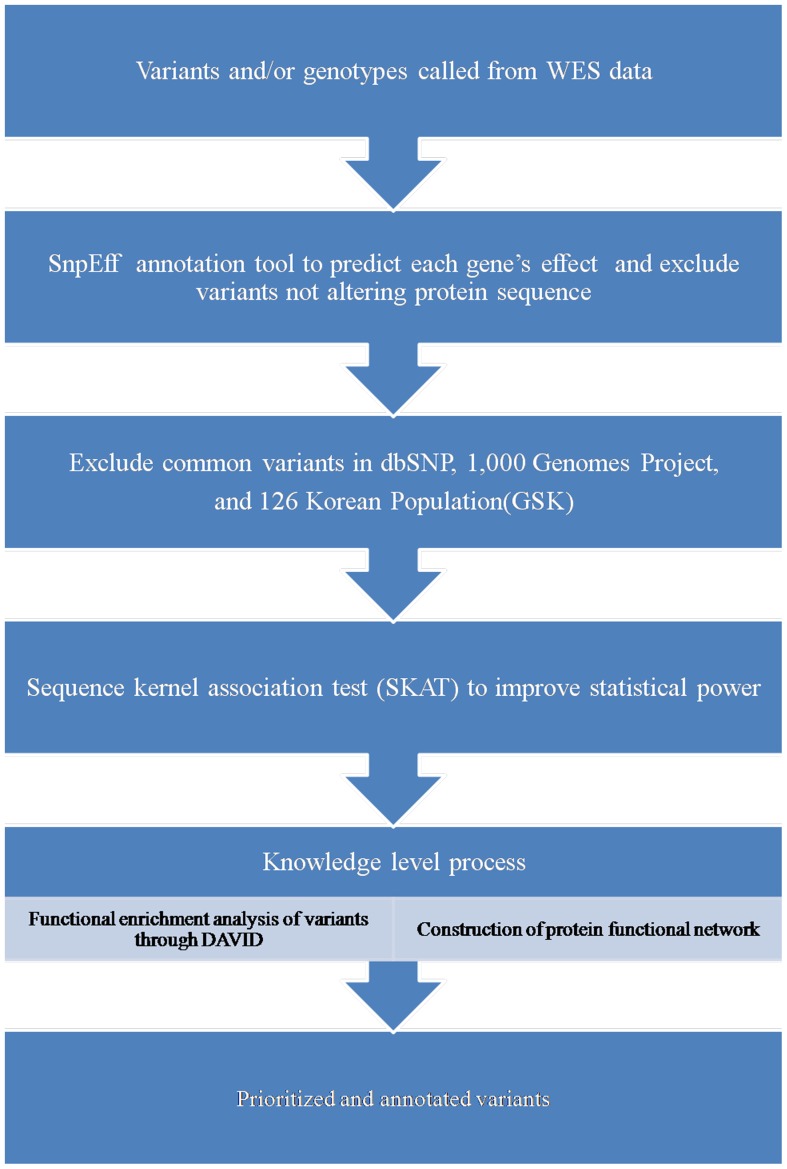
The filtration and prioritization framework used for data analysis.

B37 was used to build the reference genome. For prediction of variants (variant calling), only reads mapping to a unique position in the reference genome were used. Variants were identified with the Genome Analysis Toolkit (GATKv2.7-1) software, taking into account the single nucleotide polymorphisms obtained from the Single Nucleotide Polymorphism Database (dbSNP, National Center for Biotechnology Information) and from the 1000 Genomes project. Annotated, non-synonymous variants found in affected individuals were compared to variants present in the non-affected relatives. Variants present in affected individuals but not in healthy individuals were ranked based on this analysis to generate a list of candidate genes. The filtering process, which involved further bioinformatics analysis, is described in Figure 1.

### Statistical Analysis

Sequence Kernel Association Test (SKAT) was implemented to investigate candidate variants. SKAT combines squared single-variant score statistics, making it robust in its inclusion of neutral and protective variants and more powerful than pooling tests. SKAT is a flexible, computationally efficient regression approach that tests for association between variants in a region (both common and rare) and a dichotomous (i.e. case-control study) or continuous phenotype while adjusting for covariates, such as principle components, to account for population stratification [23]. SKAT, which performs region-based testing, directly conducts multiple regressions of a phenotype on genotypes for all variants in the region, adjusting for covariates [24]. SKAT results including magnitudes and directionality of the associations are based solely on estimates made from actual data sets. SKAT results were categorized by p-value.

### Gene set enrichment analysis

GSEA (DAVID Bioinformatics Resource 6.7) was performed to determine the statistical significance of gene sets recovered from SKAT analysis. DAVID (Database for Annotation, Visualization, and Integrated discovery) is a web-accessible program that integrates functional genomic annotations with intuitive graphical summaries. With the GSEA results, cluster enrichment analysis was performed to build a protein functional network. We used the following categories in DAVID: in the “Gene Ontology” section: “GOTERM BP ALL” and “GOTERM MB ALL;” in the “Protein Domains” section, “INTERPRO and “SMART.” We used the DAVID v6.7 service to compute functional enrichment for genetic variants from implant clustering failure patients (http://david.abcc.ncifcrf.gov/) [25], [26]. The genetic variants from implant clustering failure patients contained 2,022 unique proteins. DAVID recognized 2,013, which were used in subsequent DAVID functional analyses. We used terms with p-value calculated after Benjamini-Hochberg correction less than 0.05.

### Construction of protein functional network

**Figure 2:**
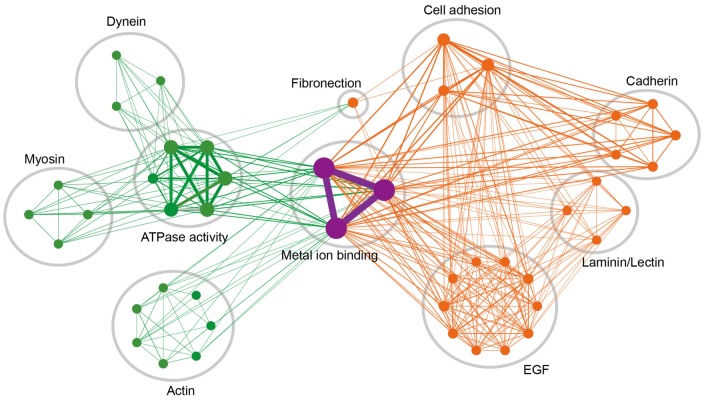
Analysis of gene function enrichment and construction of functional network. Gene functions significantly enriched in genetic variants from implant clustering failure patients are visualized as a functional cluster network. Nodes represent enriched clusters of gene functions in cell morphology (green), cell adhesion (orange), and regulation of metal ion concentration (purple). Size of nodes shows the number of genetic variants in each functional group. Edge weight is proportional to the number of shared genes. Clusters of functionally related nodes were manually circled and labeled.

The protein functional network in Figure 2 was built based on the top 10 cluster with highest enrichment scores. We linked two functional terms if they shared more than five proteins. Nodes represent enriched clusters of gene functions in cell morphology (green), cell adhesion (orange), and regulation of metal ion concentration (purple). Size of nodes shows the number of genetic variants in each functional group. Edge thickness is proportional to number of shared genes. Functional modules were manually grouped and labeled using Cytoscape 2.8 [27] (www.cytoscape.org). The labels for each node (i.e., each functional category) in Figure 2 are further explained in Table S6.

## Results

The mean age of the 6 individuals tested for WES was 59.17±7.78. A total of 55 implants were placed and 26 implants explanted from selected individuals (Table S1 in Tables S1). Quality Control (QC) run on saliva samples obtained from the six individuals confirmed samples were adequate for WES (Data S1). An average of 59,053,587 reads and 5,694 megabases were obtained from the six individual's WES results. An average of 2,886,696,571 bases was aligned with mean coverage depth of 56.04. All information regarding number of reads, sample coverage and sequencing depth, as well as the data quality, is summarized in Table S2 in Tables S1. A total of 93,955 variants were established; SnpEff, which annotates variants to eliminate ones not altering protein sequence and predicts effects of each variant on genes, narrowed variants down to 24,543. The 1000 Genomes East Asian population and Korean randomized subsamples were also used to filter out common variants by eliminating those with p-value greater than 0.05 from the two control groups. 9,243 variants were left after filtering based on the 1000 Genomes East Asian population and 5,056 after Korean randomized subsample filtering (Table 1).

**Table 1:** Number of genes common to the 6 affected individuals in each filtering step.

Filtering Step	Number of Variants	Number of Genes
Total Gene Transcripts	93,955	19,919
Impact	24,543	9,651
1000 Genomes Project	9,243	4,677
Korean randomized subsample	5,056*	3,724

*Variants were filtered based on 126 Korean individuals' alternate allele frequency >0.05

Variants were translated into genes, with a total of 3,724 gene transcripts remaining after filtering against the 1000 Genomes East Asian population and Korean randomized subsamples (Table S3 in Tables S2). SKAT analysis, which can increase statistical power when only a small number of cases are available, was applied to reveal the statistical significance of all genes (Table S4 in Tables S2). Gene transcripts were categorized by p-value. 2,022 gene transcripts remained after ranking gene transcripts by p-value (p-value <0.05). All whole exome sequencing raw data was submitted to SRA database (SRA, http://trace.ncbi.nlm.nih.gov/Traces/sra/, accession number SRP041370).

Gene set enrichment analysis (GSEA) was applied to investigate genetic variants in groups of genes sharing common biological function, domain, or pathway. We used DAVID (DAVID v6.7) to discover enriched functional-related gene groups and corrected the results using the Benjamini-Hochberg corrected p-value to correct multiple comparison errors in gene sets that can arise in WES analysis. DAVID provides a novel way to functionally analyze a large number of variants in a high-throughput fashion by classifying them into gene groups based on their annotation term co-occurrence. 927 gene sets were annotated and 175 gene sets remained after Benjamini-Hochberg corrected p-value less than 0.05. Multiple domains emerged and a few gene sets were looked into based on their roles in biological processes (Table S5 in Tables S2). GSEA detected multiple gene sets related to cell adhesion, metal ion binding, and cytoskeleton.

Protein functional network was then performed to see how genes from different domains react with each other in a biological system and how interactions between genes might affect pathogenesis of peri-implantitis. To examine the function of genetic variants from peri-implantitis patients, we searched for significantly enriched gene function clusters. Cluster enrichment was performed for the whole list of genes with mutations and the top 10 enriched clusters were picked for network visualization (Table S6) (Table 2). Clusters, terms, and genes in the protein functional network analysis were described using circular shape, nodes, and lines (Figure 2). As nodes describe terms with p-value after Benjamini-Hochberg corrected p-value of less than 0.05 and line expressed number of genes shared by each term, size of nodes shows the number of genetic variants and the thickness of the link describes the number of shared genes. Nodes represent enriched clusters of gene functions in cell morphology, cell adhesion, and regulation of metal ion concentration.

**Table 2:** Top 10 clusters ranked by enrichment score from GSEA results.

**Cadherin**	**Enrichment Score: 13.69**		
Term	Count	%	Benjamini
IPR013164:Cadherin, N-terminal	32	1.66	4.40E-13
IPR002126:Cadherin	44	2.29	4.40E-13
IPR015492:Protocadherin gamma	18	0.94	1.17E-12
**Cell adhesion**	**Enrichment Score: 12.64**		
Term	Count	%	Benjamini
GO:0007155∼cell adhesion	139	7.23	2.73E-11
GO:0022610∼biological adhesion	139	7.23	1.55E-11
GO:0016337∼cell-cell adhesion	65	3.38	1.77E-07
**EGF**	**Enrichment Score: 5.42**		
Term	Count	%	Benjamini
IPR013032:EGF-like region, conserved site	67	3.49	1.87E-07
IPR006210:EGF-like	47	2.45	4.10E-05
IPR000742:EGF-like, type 3	43	2.24	4.95E-04
**Actin**	**Enrichment Score: 3.59**		
Term	Count	%	Benjamini
IPR001715:Calponin-like actin-binding	21	1.09	0.00
IPR018159:Spectrin/alpha-actinin	12	0.62	0.01
SM00033:CH	21	1.09	0.02
**Metal ion binding**	**Enrichment Score: 3.58**		
Term	Count	%	Benjamini
GO:0043167∼ion binding	513	26.69	5.65E-04
GO:0046872∼metal ion binding	500	26.01	7.09E-04
GO:0043169∼cation binding	504	26.22	7.26E-04
**Laminin/lectin**	**Enrichment Score: 3.40**		
Term	Count	%	Benjamini
IPR001791:Laminin G	15	0.78	0.01
IPR013320:Concanavalin A-like lectin/glucanase	19	0.99	0.04
SM00282:LamG	15	0.78	0.02
**ATPase activity**	**Enrichment Score: 3.17**		
Term	Count	%	Benjamini
GO:0032559∼adenyl ribonucleotide binding	210	10.93	2.81E-04
GO:0030554∼adenyl nucleotide binding	217	11.29	6.06E-04
GO:0005524∼ATP binding	205	10.67	5.67E-04
**Fibronectin**	**Enrichment Score: 2.96**		
Term	Count	%	Benjamini
IPR003961:Fibronectin, type III	37	1.93	0.02
IPR008957:Fibronectin, type III-like fold	33	1.72	0.09
SM00060:FN3	37	1.93	0.09
**Dynein**	**Enrichment Score: 2.69**		
Term	Count	%	Benjamini
IPR013602:Dynein heavy chain, N-terminal region 2	8	0.42	0.04
IPR004273:Dynein heavy chain	8	0.42	0.04
IPR011704:ATPase associated with various cellular activities	8	0.42	0.04
**Myosin**	**Enrichment Score: 2.36**		
Term	Count	%	Benjamini
IPR000048:IQ calmodulin-binding region	23	1.20	0.01
IPR001609:Myosin head, motor region	14	0.73	0.01
SM00242:MYSc	14	0.73	0.02

Network analysis showed metal ion binding in the middle of all clusters and terms, indicating ion binding as a common factor in genes of cell adhesion and cytoskeleton ontology. Cell adhesion and cytoskeleton clusters, which affect cell morphology, showed mutations. In addition, the cytoskeleton cluster is functional in cell morphogenesis, involving dynein, myosin, actin, and ATPase activity. Clusters shared multiples genes through the protein functional network and all clusters showed interactions through metal ion binding.

We also applied the IntPath pathway integration database [28] and found that DAVID and IntPath provided equivalent results (Table S7).

Genes known to be associated with peri-implantitis and periodontitis were tested for their presence in our case group; IL-1A, IL-1B, and TNF all appeared in our dataset (Table 3) (Table S8).

**Table 3:** Known Implantitis Genes Found in All Variants.

Gene	Transcript	Variant Sample Position	Samples Affected	Samples Heterozygous
IL6	ENST00000404625	exp1_Idx_2:7:22771039	1	1
IL1B	ENST00000416750	exp6_Idx_12:2:113590977	1	1
IL1B	ENST00000418817	exp6_Idx_12:2:113590977	1	1
IL1B	ENST00000432018	exp6_Idx_12:2:113590977	1	1
IL1B	ENST00000263341	exp6_Idx_12:2:113590977	1	1
IL1A	ENST00000263339	exp3_Idx_3:2:113537072	1	1
IL6	ENST00000258743	exp1_Idx_2:7:22771039	1	1
IL6	ENST00000407492	exp1_Idx_2:7:22771039	1	1
TNF	ENST00000449264	exp5_Idx_11:6:31543574	1	1
IL6	ENST00000401630	exp1_Idx_2:7:22771039	1	1

## Discussion

With the development of different types of genome sequencing and associated analytical tools, biomarker discovery has emerged as a major area of research throughout medicine. In 2009, four individuals with Freeman-Sheldon syndrome (FSS), a rare autosomal dominant disorder, were exome-sequenced to prove that such sequencing could identify causal genetic variants [29]. As WES is known to sequence thousands of functional genes at a time, it has become the tool of choice for discovering causative genetic variants, especially in Mendelian diseases [30]. Although exome sequencing is often used to investigate Mendelian disorders, it also expands our knowledge of novel mutations of established genes linked to a particular disorder such as celiac disease and helps to uncover the complex interplay between modifier variants that contributes to a disease phenotype. For these reasons, WES has been widely used to discover genetic factors related to a range of diseases in various medical fields, particularly for diseases that exhibit broad genetic or phenotypic heterogeneity [31]–[33].

The WES approach held promise for our current research given the rarity and distinct phenotype of clustering failure in peri-implantitis. Limitations of the current study include a case population too small for generalization about the whole Korean population, linking pathogenesis of peri-implantitis solely to genetic causalities while peri-implantitis is multi-factorial disease, and use of saliva samples over blood. The saliva sample collection method was chosen based on previous studies confirming its suitability for genetic sequencing and the high percentage of case recruitment [34], [35]. After careful consideration, a WES approach was chosen for the current study, whose results showed a large set of variants, necessitating filtering to discover genetic variants specifically related to peri-implantitis. WES annotations from the 6 individuals in this study revealed a number of variants for further analysis. Standard methods used to test a disease such as peri-implantitis for association with a single common genetic variant are underpowered given the insufficient sample or effect sizes [36], [37]. Hence, the sequence kernel association test (SKAT) was used to accommodate our sample size and to improve statistical power.

SKAT results were further subjected to GSEA study via DAVID and various domains were discovered. The GSEA result was then compared to those of IntPath for confirmation using another bioinformatics approach. Adhesion between cells and implant in the long term involves regulation of aspects of cell expression such as cell membrane proteins, ECM proteins, integrins and cytoskeleton proteins, all which work together to maintain the proper adhesion. The external faces of focal contacts present specific receptor proteins. Cadherins, which showed highest association in this study, are a class of type-1 transmembrane proteins which play an important role in cell adhesion, forming adherence junctions to bind cells within tissues together and also participating in implant-bone adhesion. ECM in intracellular fluid is another important component for cell adhesion. Integrins are a major family of cell surface-adhesion receptors that can mediate cell-cell, cell-matrix, and cell-pathogen interactions. Most integrins are not intrinsically active and are often expressed on the cell surface in an inactive state, neither binding ligands nor signaling. Metal ion Ca^2+^ plays an important role in keeping integrins inactive, removal of Ca^2+^ or addition of Mn^2+^ remarkably increasing binding affinity and adhesiveness of almost all intergrins. Integrin-mediated adhesion and signaling events are important in normal physiological responses such as immune response, tissue morphogenesis, wound healing, hemostasis, cell survival, and cell differentiation [38]. Conversely, dysregulation of integrins is involved in the pathogenesis of many diseases, including cancer metastasis, auto-immune disease, and thrombotic vascular diseases. It may be assumed that insufficient metal ion concentration will cause decreased cell-to-titanium adhesion during the healing stage of implant, resulting in inflammatory disease. Also, some integrins are able to bind several ligands such as laminin and fibronectin [39]. The FN domain, a major glycoprotein in the extracellular matrix, binds specifically to titanium implants, which can serve as a ligand for a dozen members of the integrin receptor family [40]. It was reported that FN III 7–10 and FN III 9–10 synthetic peptides are effective in osteoblast adhesion, necessary for successful dental implant outcome [41]. It was also reported that FN coatings on titanium implants were advantageous for peri-implant bone formation [42]. Another high-ranking gene cluster was the Epidermal Growth Factor (EGF) domain, critical in stimulating cellular proliferation, differentiation, and survival [43]. Consolaro et al concluded that EGF in the saliva and in the epithelial cells stimulates peri-implant epithelial proliferation, thereby triggering the formation of the peri-implant junctional epithelium [44]. Remarkably, all high ranking domains in the study play a critical role in adhesion of cells to titanium surface and in the development of peri-implantitis. In the last ten years, implantology research has focused on the analysis of peri-implant fluids with the basic aims of identifying potentially valid biochemical and immunological markers of inflammatory processes and their levels and/or of predicting risk for the onset of peri-implant disease. There have been several genetic studies of the role of cytokines in peri-implantitis [18]. Cytokines are hormonal regulators or signaling molecules of host responses to infection, immune responses, inflammation, and trauma, making them significant in peri-implantitis, which broadly results from an unregulated host inflammatory response to antigen bacterial determinants from dental plaque. Clinical research indicates that monitoring the dynamics of local cytokine levels during peri-implantitis, together with research into gene polymorphism for these cytokines and other genes involved in the inflammatory process, is a valid means of achieving new methods of diagnosis, prognosis and treatment of peri-implantis. A recent systematic review on the association between genetic predisposition and dental implant biological complications implied that there is no strong association between specific genetic polymorphism (IL-1, IL-2, IL-6, TNF-α or TGF-β1) and dental implant failure in terms of biological complications although there was a potential link between the IL-1 genotype and peri-implantitis [45]. Genes known to be associated with peri-implantitis were also tested for our case group but did not show significant results, suggesting the existence of undiscovered variants linked with peri-implantitis.

The current study chose six patients with cluster failure to investigate genetic variants involved with peri-implantitis. Cluster failure is a technical term used to describe the phenomenon in which a few individuals have a concentrated risk for multiple implant failure and subsequently experience multiple losses. Among individuals sharing relatively similar environmental factors, cluster failure has low occurrence among the total population, suggesting its association with genetic factors rather than behavioral ones such as smoking, stress, or maintenance of dental implant after surgery [46]. The patients selected for the current study had severe peri-implantitis leading to implant failure with the number of occurrences varying among the individuals. The current study is limited in having selected a case population with the exclusion of environmental factors known to affect peri-implantitis. Under similar environmental influence, the distinctive phenotypes of the six patients with clustering failure were very rare among implant patients, indicating that such clustering failure was likely to be caused by individual genetic differences. With these limitations in mind, a case population with clusterization was chosen for genetic analysis. WES was thus applied in the current study to detect pathogenic genes associated with peri-implantitis in these patients experiencing clustering failure. Following WES and statistical analysis, a bioinformatics study was conducted to reveal possible pathogenic genes associated with peri-implantitis. Referencing the work of Zhou and Wong [47], which provides a thorough description of the well-known functional association database STRING, which this study initially used for functional network analysis. As the use of STRING yielded insufficient data, we built an independent functional network to investigate the relationships among functions.

Other risk factors linked with peri-implantitis include smoking, stress, diabetes, osteoporosis, and genetics. Due to the multiple causalities of peri-implantitis, patient recruitment for the current study was relatively difficult. As environmental factors such as smoking behavior can greatly affect the occurrence of peri-implantitis [48]–[50], careful consideration was given eliminating patients with environmental risk factors. Also, a randomized subsample of 126 Koreans was chosen with exclusion of disease that could affect peri-implantitis such as diabetes and osteoporosis though monitoring of other risk factors in this randomized subsample group was limited. Machalowicz et al indicated that genetics constituted the most important factor influencing differences in periodontal disease, based on studies involving twins. Moreover, that study characterized individuals varying from one another because of differences in genetic makeup and environment, as if variances in the population for a given measure could simply be partitioned into genetic and environmental causes [51]. This study focused on choosing individuals experiencing severe peri-implantitis with relatively similar environmental factors and showing a distinctive phenotype for the sake of genetic association. The small study population recruitment limits concrete conclusions. To fully elucidate the effect of environmental and genetic variances in peri-implantitis, WES results for each case population could be compared to family members without the indication of peri-implantitis.

## Conclusions

Previous analyses of peri-implant fluids aimed at identifying potentially valid biochemical and immunological markers of inflammatory processes have focused on cytokines. However, our results suggest that various genes and gene sets related to factors involved in cell adhesion such as cadherin, fibronectin, integrins, EGF domains, and cytoskeletons play critical roles in the osseointegration and pathogenesis of peri-implantitis. Interestingly, these two gene sets are indirectly linked via the metal ion binding protein. One may conclude that regulatory imbalance in metal ion concentration elicits dysfunction in cell morphology and cell adhesion, eventually causing peri-implantitis. The discovery of metal ions in this protein functional network study implies dysregulation of integrins, which could affect surface adhesion, and, subsequently, the occurrence of peri-implantitis. Genetic diagnosis before implant surgery would highlight those genes related to peri-implantitis and help clinicians to determine appropriate treatment. Also, the pathogenesis of peri-implantitis can be investigated through genetic research similar to the current study to generate tailored treatment options. Further research with more cases and controls, along with functional animal studies, may yield legitimate biomarkers for early diagnosis of peri-implantitis. Data from this research will be used to develop analytic methods for small case-only samples.

## Supporting Information

Tables S1 **This file contains Table S1 and S2.** Table S1. Patient Information. Table S2. Summary of number of reads and coverage.
(DOCX)

Tables S2 **This file contains Table S3, S4 and S5.** Table S3. Full list of genes. Table S4. Full SKAT results. Table S5. Full GSEA (DAVID) results.
(XLSX)

Table S6 **Full cluster enrichment list.**
(XLSX)

Table S7 **IntPath results.**
(XLSX)

Table S8 **Full Known Implantitis Genes Found in All Variants.**
(DOCX)

Data S1 **QC results, sequence, alignment, variants, effects counts, and genes summary.**
(XLSX)
