# Supplementary material for: Investigation of Pathogenic Genes in Peri-Implantitis from Implant Clustering Failure Patients: A Whole-Exome Sequencing Pilot Study
Source: PLoS One. 2014 Jun 12;9(6):e99360. doi: 10.1371/journal.pone.0099360 (PMC4055653; doi:10.1371/journal.pone.0099360)
Supplement: Tables S1 — This file contains Table S1 and S2. Table S1. Patient Information. Table S2. Summary of number of reads and coverage. (DOCX) [file pone.0099360.s001.docx]

**Supplementary Table**

**Table S1. Patient Information**

| Gender | Age | Number of implants placed | Number of implant failures | History of systemic diseases |
| --- | --- | --- | --- | --- |
| Female | 67 | 14 | 7 | Penicillin allergy |
| Female | 68 | 11 | 4 | None |
| Female | 53 | 6 | 4 | None |
| Male | 54 | 6 | 2 | Hepatitis |
| Female | 63 | 6 | 2 | None |
| Female | 50 | 12 | 7 | None |

**Table S2. Summary of number of reads and coverage**

| Lane | Sample ID | Index | Yield (Mbases) | # Reads | % of >= Q30 Bases (PF) | Mean Quality Score (PF |
| --- | --- | --- | --- | --- | --- | --- |
| 1 | exp1_Idx_2 | 2 | 5,428 | 53,743,622 | 89.18 | 35.02 |
| 1 | exp2_Idx_10 | 10 | 5,689 | 56,329,914 | 89.38 | 35.1 |
| 1 | exp3_Idx_3 | 3 | 7,206 | 71,350,078 | 89.14 | 35.04 |
| 1 | exp4_Idx_9 | 9 | 4,624 | 45,781,248 | 89.36 | 35.08 |
| 1 | exp5_Idx_11 | 11 | 5,964 | 59,049,854 | 88.85 | 34.92 |
| 1 | exp6_Idx_12 | 12 | 6,875 | 68,066,808 | 89.53 | 35.14 |

**(All below are in separate excel file)**

**Table S3. Full list of genes**

**Table S4. Full SKAT results**

**Table S5. Full GSEA (DAVID) results**

**Table S6. Full cluster enrichment list**

**Table S7. IntPath results**

**Supplementary Figure**

**Figure S1. QC result**
