# Supplementary material for: Investigation of Pathogenic Genes in Peri-Implantitis from Implant Clustering Failure Patients: A Whole-Exome Sequencing Pilot Study
Source: PLoS One. 2014 Jun 12;9(6):e99360. doi: 10.1371/journal.pone.0099360 (PMC4055653; doi:10.1371/journal.pone.0099360)
Supplement: Table S8 — Full Known Implantitis Genes Found in All Variants. (DOCX) [file pone.0099360.s005.docx]

**Table 3. Known Implantitis Genes Found in All Variants**

| Gene | Transcript | Variant Sites Count | Variant Count | Variant Sample Position | Samples Affected | Samples Heterozygous | Samples Homozygous Alternate Allele | Samples Homozygous Reference |
| --- | --- | --- | --- | --- | --- | --- | --- | --- |
| IL6 | ENST00000404625 | 1 | 1 | exp1_Idx_2:7:22771039 | 1 | 1 | 0 | 5 |
| IL1B | ENST00000416750 | 1 | 1 | exp6_Idx_12:2:113590977 | 1 | 1 | 0 | 5 |
| IL1B | ENST00000418817 | 1 | 1 | exp6_Idx_12:2:113590977 | 1 | 1 | 0 | 5 |
| IL1B | ENST00000432018 | 1 | 1 | exp6_Idx_12:2:113590977 | 1 | 1 | 0 | 5 |
| IL1B | ENST00000263341 | 1 | 1 | exp6_Idx_12:2:113590977 | 1 | 1 | 0 | 5 |
| IL1A | ENST00000263339 | 1 | 1 | exp3_Idx_3:2:113537072 | 1 | 1 | 0 | 5 |
| IL6 | ENST00000258743 | 1 | 1 | exp1_Idx_2:7:22771039 | 1 | 1 | 0 | 5 |
| IL6 | ENST00000407492 | 1 | 1 | exp1_Idx_2:7:22771039 | 1 | 1 | 0 | 5 |
| TNF | ENST00000449264 | 1 | 1 | exp5_Idx_11:6:31543574 | 1 | 1 | 0 | 5 |
| IL6 | ENST00000401630 | 1 | 1 | exp1_Idx_2:7:22771039 | 1 | 1 | 0 | 5 |
